# Dietary protein splanchnic uptake and digestibility via stable isotope tracers

**DOI:** 10.1097/MCO.0000000000001061

**Published:** 2024-07-24

**Authors:** Jake Cox, Daniel James Wilkinson, Philip James Atherton, Kenneth Smith

**Affiliations:** Centre of Metabolism, Ageing and Physiology, University of Nottingham, Royal Derby Hospital Medical School, Derby, UK

**Keywords:** amino acids, dietary protein, digestibility, skeletal muscle, stable isotope tracers

## Abstract

**Purpose of review:**

Dietary proteins are broken down into peptides across the gastrointestinal tract, with skeletal muscle being a primary deposition site for amino acids in the form of incorporation into, for example, metabolic and structural proteins. It follows that key research questions remain as to the role of amino acid bioavailability, of which protein digestibility and splanchnic sequestration (absorption and utilization) of amino acids are determining factors, impact upon muscle protein synthesis (MPS) in clinical states.

**Recent findings:**

Elevated splanchnic amino acid uptake has been implicated in anabolic resistance (i.e. attenuated anabolic responses to protein intake) observed in ageing, though it is unclear whether this limits MPS. The novel ‘dual stable isotope tracer technique’ offers a promising, minimally invasive approach to quantify the digestion of any protein source(s). Current work is focused on the validation of this technique against established methods, with scope to apply this to clinical and elderly populations to help inform mechanistic and interventional insights.

**Summary:**

Considerations should be made for all facets of protein quality; digestibility of the protein, absorption/utilization and subsequent peripheral bioavailability of amino acids, and resultant stimulation of MPS. Stable isotope tracer techniques offer a minimally invasive approach to achieve this, with wide-ranging clinical application.

## INTRODUCTION

Stable isotope tracers offer the opportunity to study mechanistic aspects of protein digestibility. For instance, the quantification of splanchnic amino acid extraction using stable isotope tracers is important to differentiate the roles played by amino acid in both splanchnic and systemic metabolic processes in individual organs/tissues and to understand whole body protein and amino acid requirements [1]. Secondly, the recently developed ‘dual isotope tracer’ technique (please refer to ensuing sections) facilitates assessment of the extent to which distinct protein sources/clinical conditions impact the digestion of protein, crucially, using any protein-containing foodstuffs [2^▪▪^], that is, not being limited to intrinsically labelled protein sources. Digestibility is often used to describe the digestion of a protein source to its constituent amino acids, which should be measured within the small intestine, that is, the ileum, but is sometimes also inferred from the appearance of individual amino acid within the systemic circulation following a protein feed. The ‘true digestibility’ of a protein source can be assessed using the naso-ileal sampling approach, or more recently using the dual tracer approach, both of which are described in the following. Simply measuring the appearance of amino acid in the systemic circulation, reflects the bioavailability of amino acid to peripheral tissues and organs, but does not take account of splanchnic uptake and utilization of amino acid across the splanchnic tissues. We will use digestibility to mean the digestion of protein to its constituent amino acid.

As the principle storage reservoir for amino acid in the form of proteins, muscle proteostasis is significantly impacted by amino acid bioavailability, which is in turn determined by the digestibility of a protein source coupled to the splanchnic extraction of amino acid. The extent to which amino acid bioavailability influences postprandial MPS is currently unclear. This was recently summarized in a review by Horstman and Huppertz [3^▪^], which included studies assessing both postprandial aminoacidemia and MPS rates in response to a range of protein sources and hydrolysis; showing protein sources that produce significantly greater aminoacidemia only sometimes result in significantly greater MPS. Under conditions of anabolic resistance such as in aging, enhancing this postprandial amino acid bioavailability may be more pertinent to ensure a maximal MPS responses [4^▪^]. This review will as such focus upon stable isotope tracer techniques in the quantification of splanchnic amino acid uptake and digestion of proteins and their most recent clinical applications. 

**Box 1:**
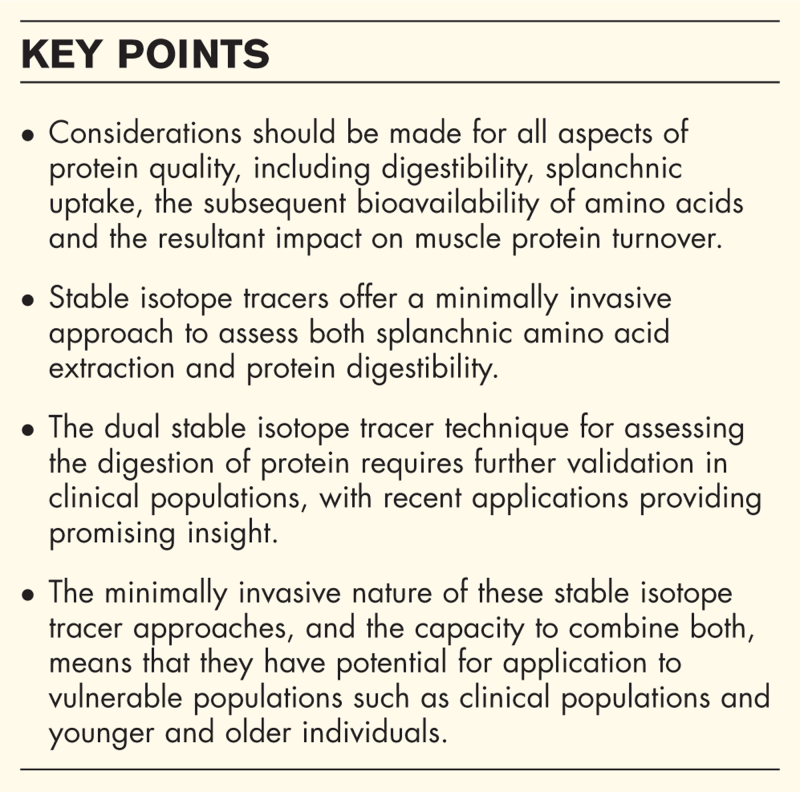
no caption available

## DETERMINATION OF PROTEIN DIGESTIBILITY USING STABLE ISOTOPES

**FIGURE 1:**
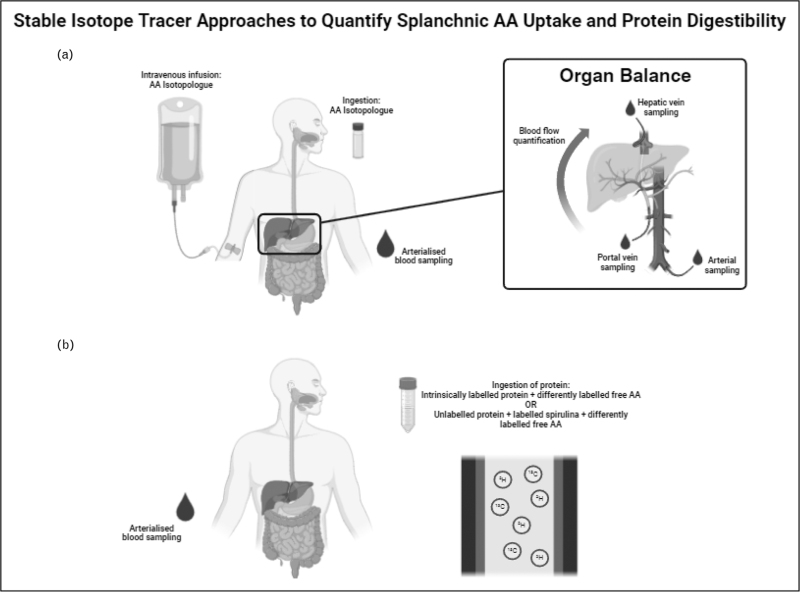
(a) Simultaneous administration of intravenous and oral stable isotopically labelled amino acid (incorporating different isotopes e.g. ^2^H-AA intravenously, ^13^C-AA orally or multiply labelled ^2^H_2_-AA intravenously, ^2^H_5_-AA orally) to measure splanchnic amino acid uptake. This is achieved from measuring the rate of appearance of both tracers in arterialized blood using a single-pool model. Splanchnic amino acid uptake is calculated based on the relative plasma enrichments from oral and intravenous stable isotope tracers, as follows: $Splanchnic\;Extraction=1-\left[\left(E_{oral}/I_{oral}\right)/\left(E_{iv}/I_{iv}\right)\right]$ where *E*_*oral*_ is the plasma enrichment from the oral tracer, *I*_*oral*_ is the infusion rate of the oral tracer, *E*_*iv*_ is the enrichment of the intravenous tracer and *I*_*iv*_ is the infusion rate of the intravenous tracer. Additionally, sampling from the hepatic portal vein, and femoral vein and artery permits application of the organ balance approach to quantify AA uptake and protein turnover in the liver, as follows: balance = (*C*_A_ – *C*_V_) ∗ BF. Breakdown = *E*_A_/*E*_v_ – 1 ∗ *C*_A_ ∗ BF. Synthesis = breakdown + balance. where *C*_A_ and *C*_v_ are arterial and venous amino acid concentrations, respectively, BF is organ blood flow and *E*_A_ and *E*_V_ are arterial and venous amino acid enrichments, respectively. (b) The dual stable isotope tracer approach to quantify protein digestibility, which can be calculated using an intrinsically labelled protein source or and unlabelled protein source combined with labelled protein (e.g. U-^13^C spirulina), and either differently labelled free amino acid (as incorporated in the intrinsically labelled protein), or a universally labelled amino acid mixture (U-^2^H free amino acid mix). Digestion is calculated based upon the ratio of plasma amino acid enrichments derived from the intrinsically labelled protein, or combined unlabelled/spirulina protein mixture with reference to the free labelled amino acid (which are assumed to represent 100% bioavailability), as follows: digestibility = [plasma ^13^C-AA (APE)/meal ^13^C-AA (APE)]/[plasma ^2^H-AA (APE)/meal ^2^H-AA (APE)] ∗ 100.

To study splanchnic protein metabolism, extraction of amino acids can be estimated through the simultaneous application of intravenous and intragastric stable isotopically labelled amino acids (^13^C, ^2^H, or isotopologues – multiply labelled, i.e. ^2^H_2_, ^2^H_5_, 1-^13^C or U-^13^C amino acids) as shown in Fig. 1a, that is, where the relative rate of appearance of intravenously delivered amino acids compared with orally derived amino acids reflects the fraction taken up by the splanchnic bed. The ‘organ balance’ tracer technique expands upon this, requiring sampling from the femoral artery, femoral vein and hepatic vein to quantify net amino acid balance, that is, uptake and or release, by specific organs such as the liver and the gut, as well as providing measures of protein synthesis and breakdown, via inclusion of isotopes and dilution measurements combined with net balance measures. However, this approach is challenging in requiring catheterization of an artery for sampling and the vein immediately distal to the organ of interest, as well as a measure of the rate of blood flow across the organ (albeit which can be achieved by different techniques such as indocyanine green dye, Doppler flowmetry, xenon computed tomography and MRI). Notably, to quantify amino acid uptake by the liver, catheterization of the hepatic portal vein is also required, as per Fig. 1a.

Historically, true digestibility of a protein source has been quantified *in vivo* by sampling the terminal ileum. Consequent to the invasiveness, pig models are most often used. Despite producing robust data, this is not a sustainable approach for the broad range of individual protein sources and protein blends that exists, due to the invasiveness of the technique. The recent development of the dual stable isotope tracer approach for measuring the digestion of dietary protein offers a less-invasive alternative that can be easily applied in humans [5^▪^]. For a full description of the methodology associated with the dual stable isotope tracer technique, readers are directed to the recent methodological review from Kashyap *et al.*[2^▪▪^]. Briefly, this involves a plateau (steady-state) feeding protocol, which includes any protein source(s), which can be of any matrix of foodstuffs and may be either unlabelled or intrinsically labelled protein, that is compared with a uniformly labelled test protein, for example, U-^13^C-spirulina (as long as any intrinsically labelled amino acid are distinguishable to the U-^13^C labelled amino acid, usually a mass difference (e.g. leucine molecular weight = 131.17 g/mol, U-^13^C leucine molecular weight = 132.17 g/mol)). Labelled free amino acids (individual amino acid or a mix, e.g. uniformly labelled ^2^H free amino acid), that can be differentiated by mass spectrometry, are given in the meal and are assumed to represent 100% breakdown of the protein, as shown in Fig. 1b. The ratio of enrichment of the amino acid from the test protein (or intrinsically labelled protein) and the enrichment of the labelled free amino acid can then be used to assess protein digestibility. Alternatively, the intrinsically labelled or test protein meal and labelled free amino acid can be provided as a bolus and a nonsteady-state approach used, for example, Steele equations. The primary advantage of this technique is the minimally invasive nature, requiring only blood sampling, opening application to vulnerable populations such as the aged, frail, and clinical populations.

## NASO-ILEAL VERSUS DUAL TRACER APPROACHES FOR ESTIMATING PROTEIN DIGESTION

Only two studies thus far have simultaneously compared the digestibility values obtained for protein sources using both the naso-ileal sampling method and the dual stable isotope tracer approach [6^▪^,^7^^▪▪^]. For casein, a slow digesting protein source, the dual stable isotope tracer approach significantly underestimated digestibility compared with ileal sampling for most of the essential amino acids (EAAs) assessed, whereas with pea protein, also a relatively slow digesting protein, the dual stable isotope approach, under- (Lys/Phe), over- (Ile/Thr) and gave similar estimates (Leu/Val) to the naso-ileal method [7^▪▪^]. It is important to consider that due to the emerging nature of this methodology, only three different protein sources have insofar been assessed, and that the use of ^15^N-labelled sunflower protein isolate where transamination and reamination pathways across the splanchnic bed may have impacted the dual tracer approach; nonetheless, both Threonine and Lysine amino acids, which do not undergo transamination across the splanchnic area showed around 10–15% lower uptake [6^▪^].

Taken in combination with other studies [8^▪^], which produced comparable data for red kidney bean protein using the dual stable isotope tracer approach to previous ileal digestibility animal models, these data suggest there is inherent value in applying this method owing to the apparent accuracy and simplicity of the approach. In contrast, somewhat lower values for fava beans in humans [9^▪^] was reported than in animal models. Using the naso-ileal sampling technique in humans, an average indispensable amino acid digestibility of 90 ± 6% for fava bean protein [10^▪^], compared with 61 ± 5% reported using the dual stable isotope tracer technique [9^▪^]. Some of these differences may be attributed to the relatively low sample size of five participants in the study by Khayour *et al.*[9^▪^], as well as variations in food preparation techniques employed across both studies. The minimally invasive nature of the approach, particularly when using U-^13^C spirulina as test protein (avoiding the challenge in generating intrinsically labelled protein), engenders the dual method appealing, and the method of choice for use in clinical and frail populations. Clearly, further studies are needed to validate the efficacy and utility of this technique in clinical conditions where nutritional need and requirements are not fully understood, possibly in tandem with measures of splanchnic uptake and utilization.

## CLINICAL APPLICATIONS OF STABLE ISOTOPES TO DIGESTIBILITY: AGEING, TO CRITICAL CARE

Excessive splanchnic uptake of amino acids has been implicated in anabolic resistance of ageing [4^▪^,11^▪^], whereby reduced systemic bioavailability of amino acids may limit postprandial muscle protein synthesis, which over time, contributes to sarcopenia. Reflecting this, the simultaneous intravenous and intragastric stable isotope tracer approach to assess splanchnic extraction has suggested that EAA extraction, that is, Leu and Phe, is higher in older than young men, although this did not result in reduced muscle protein synthesis in older individuals [1]. Apparent discrepancies between studies in the relationship between splanchnic amino acid extraction and plasma amino acid availability likely relate to the protein doses consumed; once the splanchnic tissues have utilized what amino acid they require for energy, intermediary metabolism and protein synthesis, the excess escapes the splanchnic bed, whereby subsequent utilization and clearance will be determined by body cell mass, muscle protein turnover, and further intermediary metabolism. Therefore, it may be that splanchnic extraction as a percentage of the protein dose may be greater when the protein dose is lower, and that this can be exacerbated in situations when amino acid demand is increased across the splanchnic bed, particularly by the liver, for example, during the acute phase response, and to changes in splanchnic extraction that may occur during an increased immune response, given that these cells use nonessential amino acids (NEAAs) as a primary fuel source, and for replication. It is well established that the majority of splanchnic amino acid uptake consists of NEAAs, particularly glucogenic amino acids, such as glutamate, some 80% of glutamate is sequestered across the splanchnic bed, glutamine, and alanine. By contrast, the liver lacks branched chain aminotransferase 2 for transamination of branched chain amino acids (BCAAs) to branched chain alpha-keto acids, meaning that approximately 75% of BCAAs escape the splanchnic bed for utilization by peripheral tissues, such as skeletal muscle [12^▪^] (slightly more, 40–50%, of Phenylalanine and Arginine have been shown to be sequestered by the splanchnic bed). It is clear EAAs and NEAAs are handled differently by the splanchnic bed and that the total dose may be important in determining the relative proportion of each amino acid that escapes the splanchnic bed. NEAA supply is not limiting to the periphery given the metabolic role muscle plays in storing NEAAs, for example, 20 mmol/l Gln and providing NEAA to the rest of the tissues in the fasted and fed states, that is, net release of Glu, Gln, and Ala. Robust measures of protein digestion, splanchnic extraction, and amino acid utilization are required to understand the complex interplay of these processes in health, ageing, and disease. This may be possible by combining these approaches simultaneously, with appropriate choice of intravenous, intragastric tracers, and intrinsically labelled protein (or U-^13^C or U-^2^H spirulina test protein).

The recent literature is sparse when it comes to protein/amino acid needs during critical illness, which represents the most rapid form of muscle wasting. Previous research has suggested that in critical illness, muscle protein synthesis responsiveness to feeding is reduced and protein breakdown is elevated, through the reduced sensitivity to the anticatabolic effect of insulin — both of which serve to increase circulating amino acid levels possibly to meet the demands of the liver for gluconeogenesis, production of acute phase proteins, and for substrates for immune cell production. Theoretically, it could follow that there may also be enhanced splanchnic amino acid uptake under such conditions to further meet these demands. A recent study assessed the splanchnic uptake of phenylalanine in critically ill patients, on mechanical ventilation, using a combined intravenous infusion of ^2^H_5_-phenylalanine with intraduodenal feeding of 20 g of intrinsically 1-^13^C-phenylalanine labelled milk protein [13^▪^]. However, they reported no significant differences between healthy controls and critically ill, in the percentage of exogenous protein-derived amino acids appearing in the systemic circulation (54 ± 9 and 62 ± 13%, respectively). Also, the reported appearances of systemic phenylalanine was within the range of splanchnic extraction reported in the historical literature (29–50%).

There is evidently a need for further investigation into the effects of different health conditions, particularly metabolic disorders, and the role of protein nutrition, on splanchnic amino acid uptake, which may provide insights into the true extent to which the splanchnic bed impacts, splanchnic health, and systemic amino acid availability and by default the regulation of muscle protein synthesis in clinical conditions. Therefore, although these findings provide interesting insights into the splanchnic uptake of amino acid in clinical settings; given there is evidence that nutritional status (fasted versus fed), dose and source of protein can impact the level of first pass splanchnic uptake and amino acid availability in the periphery, in addition to age and underlying health status, all effect digestibility, splanchnic uptake, and amino acid bioavailability, controlled studies are needed to understand the importance of these processes on health and disease.

### Will isotope protein digestibility studies have an impact in clinical settings?

The recent focus on technical validation, along with the requirement for sensitive measures of isotope labelling by isotope ratio mass spectrometry, may explain the paucity of current studies using the dual stable isotope tracer approach to quantify digestibility of different protein sources in clinical or elderly populations, despite the apparent promise, simplicity, and practicability of the approach. In assessing a range of high-quality (i.e. whey, casein) and low-quality (i.e. plant-based sources) proteins, insights can be provided on the efficacy of these different sources in a range of disease states. A particular focus should perhaps be on gastrointestinal diseases, given that protein malabsorption is potentially prevalent in these conditions, and many gastrointestinal diseases are also associated with a high prevalence of sarcopenia [14^▪^,15^▪^], with muscle being the major depot of postprandial amino acid storage in the form of protein. To date, only one study has applied the dual tracer approach to measure this in a clinical setting, showing that individuals with cystic fibrosis had less than half the digestive capacity of healthy individuals, which increased to 90% with the intake of pancreatic enzymes [16]. This highlights the validity of the dual tracer technique both for quantifying protein digestibility in clinical populations and for assessing the role of intervention strategies to enhance amino acid availability.

Further, there may be some capacity for food processing techniques such as cooking and heat processing to improve the availability of amino acid from protein sources [17^▪^], which could prove beneficial when applied to clinical populations. This has been demonstrated already using the dual stable isotope tracer technique, where dehulled mung beans were reported to have increased amino acids derived from protein (∼8% higher), when compared with whole mung bean [18]. Not only does this highlight the potential for food processing techniques to improve protein-derived amino acid availability, which may benefit clinical populations where dietary protein use is compromised but also further validates the sensitivity and applicability of the dual stable isotope tracer approach, given that it was able to differentiate significant differences in digestion of the same protein source, as a result of processing alone. Future research should focus on quantifying the extent to which the digestibility of protein sources impacts on health and disease and how preprocessing of cheaper, widely available, low-quality protein sources can be utilized and combined to maximize bio-availability of amino acids to support splanchnic and whole-body individual amino acid needs.

## CONCLUSION AND FUTURE RECOMMENDATIONS

Splanchnic amino acid sequestration has been implicated in the development of sarcopenia, but there is little clarity how important this is, or if it impacts progression. The dual stable isotope tracer approach for the minimally invasive quantification of protein digestion is innovative, and has to-date focused on validation against the established naso-ileal technique. The potential application to study clinical populations, given ease of use, makes this a compelling technique to understanding the role of protein source, quality and requirements on digestion, splanchnic extraction, and subsequent systemic bioavailability. It should be possible to combine these approaches of stable isotopically labelled amino acids to measure protein digestion, splanchnic extraction, bioavailability in whole-body/muscle metabolism in a single design.

## Acknowledgements

*This work was supported by the Medical Research Council [grant number MR/P021220/1] as part of the MRC-Versus Arthritis Centre for Musculoskeletal Ageing Research awarded to the Universities of Nottingham and Birmingham and by the NIHR Nottingham Biomedical Research Centre. The views expressed are those of the author(s) and not necessarily those of the NHS, the NIHR or the Department of Health and Social Care.*

### Financial support and sponsorship

*None.*

### Conflicts of interest

*There are no conflicts of interest.*

## REFERENCES AND RECOMMENDED READING

Papers of particular interest, published within the annual period of review, have been highlighted as:

▪ of special interest
▪▪ of outstanding interest
